# Mast Cell Degranulation Exacerbates Skin Rejection by Enhancing Neutrophil Recruitment

**DOI:** 10.3389/fimmu.2018.02690

**Published:** 2018-11-20

**Authors:** Flavie Ngo Nyekel, Emeline Pacreau, Samira Benadda, Rasha Msallam, Magnus Åbrink, Gunnar Pejler, Jean Davoust, Marc Benhamou, Nicolas Charles, Pierre Launay, Ulrich Blank, Gregory Gautier

**Affiliations:** ^1^INSERM UMRS 1149, Paris, France; ^2^CNRS ERL8252, Paris, France; ^3^Université Paris Diderot, Sorbonne Paris Cité, Laboratoire D'excellence INFLAMEX, Paris, France; ^4^Institut Necker Enfants Malades, INSERM U1151, CNRS, UMR8253, Faculté de Médecine, Université Paris Descartes, Sorbonne Paris Cité, Paris, France; ^5^Singapore Immunology Network (SIgN), Agency for Science, Technology and Research (A*STAR), Singapore, Singapore; ^6^Section of Immunology, Department of Biomedical Sciences and Veterinary Public Health, Swedish University of Agricultural Sciences, VHC, Uppsala, Sweden; ^7^Department of Anatomy, Physiology and Biochemistry, Swedish University of Agricultural Sciences, Uppsala, Sweden; ^8^Department of Medical Biochemistry and Microbiology, Uppsala University, Uppsala, Sweden

**Keywords:** mast cells, neutrophils, degranulation, skin, transplantation

## Abstract

Recent evidences indicate an important role of tissue inflammatory responses by innate immune cells in allograft acceptance and survival. Here we investigated the role of mast cells (MC) in an acute male to female skin allograft rejection model using red MC and basophil (RMB) mice enabling conditional MC depletion. Kinetic analysis showed that MCs markedly accelerate skin rejection. They induced an early inflammatory response through degranulation and boosted local synthesis of KC, MIP-2, and TNF. This enhanced early neutrophil infiltration compared to a female-female graft-associated repair response. The uncontrolled neutrophil influx accelerated rejection as antibody-mediated depletion of neutrophils delayed skin rejection. Administration of cromolyn, a MC stabilizer and to a lesser extent ketotifen, a histamine type I receptor antagonist, and absence of MCPT4 chymase also delayed graft rejection. Together our data indicate that mediators contained in secretory granules of MC promote an inflammatory response with enhanced neutrophil infiltration that accelerate graft rejection.

## Introduction

Many chronic diseases have in common severe tissue destruction with solid organ or tissue transplantation often representing the unique solution to avoid end stage organ failure (1). Transplantation is now possible for many organs (heart, lung, liver, pancreas, kidneys) and tissues (skin, heart valves, cornea etc) enabled by the use of potent immunosuppressive drugs to maintain immunological tolerance (2). Yet, they are associated with severe side effects increasing tumor incidence, susceptibility to infections, and chronic graft inflammatory processes. While T cell- and Antibody (Ab)-mediated rejection mechanisms have been known for long as the prime actors (3), new evidences show that tissue inflammatory responses as well as the immunological environment including NK cells, dendritic cells, monocytes/macrophages, neutrophils, and eosinophils can importantly impact allograft acceptance and/or destabilize an established tolerant state (4–7). These findings of high clinical relevance advocate for novel strategies to minimize local inflammation in engrafted tissues.

Skin allografting is performed in case of injuries, burns, or other illnesses of the skin to overcome the loss of the skin protection. It requires an extraction of a donor skin surface area in order to transplant it onto another skin zone of the recipient. This is generally linked to a strong inflammatory and immune response that significantly compromises graft survival even in the presence of immunosuppressive treatments (8, 9). During this response, granulocytes, macrophages, T cells, and dendritic cells (DCs) infiltrate the graft releasing proinflammatory cytokines and angiogenic factors. Concomitantly, donor DCs migrate from the graft to the recipient's secondary lymphoid organs to present donor antigens and elicit an adaptive immune response (9, 10).

Mast cells (MC) are widely predominant sentinel cells in the skin (11). They contribute to host defense via the innate immune system involving many different receptors (12, 13). They also participate in adaptive immune responses protecting the host from parasites, venoms and other noxious substances via an IgE-mediated recognition (14). In allografting it has been noted early on that MC numbers increase both during acute and chronic rejection in various organs (15). Previous data showed that they represent essential intermediates in inducing tolerance in a graft protocol under tolerizing conditions. This involved their ability to support regulatory T cell function (16) by increasing tolerogenic DC in draining lymph nodes through the release of GM-CSF (17) as well as tryptophan hydroxylase-1 (Tph-1) creating a nutrient-stressed environment that favors allograft tolerance (18). Although known to accumulate in grafts, their function in transplantation under non-tolerizing conditions is poorly characterized. One study reports a protective role in a rat cardiac allograft model (19), while others did not notice significant differences in acute or chronic rejection (20). Besides rejection, MC have also been associated with ischemia reperfusion injury in various organs (21), which is known to represent a common complication in transplantation due to the prolonged ischemia time of the transplanted organ. MC are known to exert their proinflammatory action by rapidly secreting mediators from sources prestored in cytoplasmic granules by degranulation (22). These have potent effects in tissues such as the well-known effect of histamine on the vasculature or of MC-specific proteases in remodeling (23). The initial burst of degranulation is followed by the *de novo* secretion of additional mediators such as prostaglandins and leukotrienes (24) as well as a vast array of cytokines and chemokines further enhancing the inflammatory response but also fulfilling immunoregulatory functions that bias the way other immune cells behave within the inflammatory network (24, 25).

To test the implication of MCs in skin grafting, we have set up a skin graft model where C57BL6 male male-specific (H-Y) histocompatibility minor transplantation antigen donor ear skin is grafted to the ventral side of a female recipient's ear (26, 27). In this model rejection of H-Y disparate skin, besides CD8 cytotoxic T cells can also be accomplished by CD4 effector T cells, possibly through the help of antigen-nonspecific innate effector cells (28). We used our Red Mast cell and Basophil (RMB) mouse model that allows visualization and conditional depletion of MCs (29). In contrast to c-kit dependent models of MC deficiency RMB mice do not show hematopoietic abnormalities with the exception of basophils. However, basophils become rapidly replenished within 6 days, while this takes considerably more time for MCs providing a specific time window for the analysis of MCs.

Using this approach we show that MCs accelerate early graft rejection through an innate mechanisms involving their ability to enhance neutrophil mediated inflammation after degranulation within the engrafted tissues.

## Materials and methods

### Mice

C57BL/6J mice were purchased from Charles River Laboratories (L'Arbresle, France). RMB (official name, B6. Ms4a2^tm1Mal^) and *Mcpt4*^−/−^ mice have been described (29–31). In addition, RMB mice bred with Foxp3-IRES-eGFP mice (32) in the C57Bl/6J backround were used. All mice were housed under strictly controlled specific pathogen-free conditions at the mouse facilities at Bichat Medical School. In all experiments, mice were 10 to 12 weeks old. For MC depletion, RMB mice expressing the human diphtheria toxin (DT) receptor and the fluorescent protein TdTomato gene cassettes under the control of the FcεRI β-chain specifically expressed in MCs and basophils (29) were injected first i.p. with 1 μg of DT followed 2 days later i.p. plus i.v. (1 μg each) followed by a last local injection 10 days later of 100 ng DT s.c. at the base of the ear pinna. PBS-treated RMB mice were used as controls. Skin transplantation was performed 2 days later. All animal experimental protocols were approved by the French Agricultural Office and the INSERM ethics committee, and all animal care and experimentation were conducted in accordance with European directives.

### *In vivo* neutrophils depletion and drug treatments

For neutrophil depletion experiments, 200 μg of a rat anti–mouse Ly6G Ab (clone NIMP-R14) or irrelevant control rat Ab was injected twice i.p. into C57Bl/6 mice 24 h before and, at day 3 post-ear skin transplantation as previously described (33). Ketotifen fumarate (Sigma-Aldrich), a histamine H1-*receptor* antagonist, or DMSO solvent control was injected i.p. into C57Bl/6 mice at 32 mg/kg in 0.2 mL PBS 12h prior transplantation and then every day for 6 days (34). Cromolyn Sodium Salt (Sigma-Aldrich; 100 mg/kg) in 0.2 mL PBS was injected sc. 48 h, 24 h and 30 min before transplantation and then every day for 6 days to block mast cell degranulation (35).

### Ear skin transplantation

A male to female sex-mismatched minor histocompatibility H-Y antigen ear skin allograft model was performed as described (26, 27). Briefly, female (syngeneic) or male (allograft) donor mice were euthanized and a 5 × 5 mm flap of skin comprising the epidermis and dermis, but not donor cartilage, from the ventral side of the ear was removed and placed in cold saline solution. Recipient female mice were anesthetized and a 5 × 5 mm flap of skin (epidermis, dermis) from the ventral side of the ear was replaced with the female syngeneic or male allograft donor skin. We applied four stitches (8/0 Dexon, Davis and Geck) to maintain the graft. Grafts were monitored for rejection for 35 days by evaluating the necrotic surface area and rejection scores (0 = no rejection, 1 = < 25% rejection, 2 = 25–50% rejection, 3 = 50–75% rejection, 4 = 75–100% rejection, 5 = 100 % rejection) were determined.

### Flow cytometry analysis

Ears and draining cervical lymph nodes (dLN) were collected from grafted mice 2 or 6 days after skin transplantation. Ear and dLN were split mechanically and digested in RPMI 1,640 containing 1% FCS, 0.25 mg/mL of Liberase TL (Roche, Diagnostics Corp.) and 0.25 mg/mL DNase I (Sigma-Aldrich) for 90 min at 37°C. Cell suspensions were then filtrated on a 40 μm cell strainer (Falcon) in FACS buffer (PBS 2% FCS 2 mM EDTA). The following fluorochrome-conjugated anti-mouse monoclonal antibodies were used: CD11b (M1/70); Ly6C (AL-21); Ly6G (1A8); βTCR (H57-597); CD45 (30-F11); CD19 (1D3); (all from BD) F4/80 (BM8); CD117 (ACK2); and FcεRIα (MAR-1; all from eBioscience). Viability was checked by ghost violet 510 viability dye staining (Thermo Fisher) in PBS for 30 min at 4°C in the dark. After blocking Fc receptors with 2.4G2 antibodies cells were stained with Abs in PBS with 2% FCS and 2 mM EDTA for 30 min at 4°C. Cells were gated as follows: after gating out doublets, CD45+ cells were identified, then CD19+ B cells and βTCR+ T cells (CD4+ and CD8+) were gated. Among CD19– and βTCR– cells, neutrophils (CD11b+Ly6G+) were detected. In the CD19–βTCR–Ly6G– cell population, the tdT+ cells (basophils and MCs), monocytes (CD11b+Ly6G–Ly6C+) were identified. MC were tdT+FcεRIα+CD117+, and resident macrophages were CD11b+F4/80+Ly6G–Ly6Clo, as previously described (29). Fluorescent cells were detected using a BD LSR Fortessa instrument with Diva software (BD Biosciences) and analyzed using FlowJo software (Tree Star Inc.). Absolute numbers were determined following direct counting of live total cells under the microscope for each group with calculations taking into account the percentage of CD45^+^ cells.

### Cytokine quantification

Ear skin tissue around the graft area (10 × 10 mm) or whole draining lymph nodes of the ear were cut at 2 or 6 days after skin transplantation and total RNA was extracted using the Plus Mini Kit (Quiagen, Les Ulis, France). Reverse transcription was carried out using 1 μg of total RNA and Moloney murine leukemia virus reverse transcriptase (Invitrogen). For determining the quantitative transcription profile of cytokines the following primers were used: KC, forward, 5′-TCC CCA AGT AAC GGA GAA AGA A-3′, reverse, 5-‘TGT CAG AAG CCA GCG TTC AC-3′, probe, FAM-5′- AGA CTG CTC TGA TGG CAC CGT CT-3′-TAMRA, TNF, forward, 5′-CAT CTT CTC AAA ATT CGA GTG ACA A-3′, reverse, 5′-TGG GAG TAG ACA AGG TAC AAC CC-3′, probe, FAM-5′-CAC GTC GTA GCA AAC CAC CAA GTG GA-3′-TAMRA; TGF-β, forward; 5′-TGA CGT CAC TGG AGT TGT ACG G-3′, reverse, 5′-GGT TCA TGT CAT GGA TGG TGC-3′, FAM-5′-TTC AGC GCT CAC TGC TCT TGT GAC AG-3′-TAMRA; IL6, forward, FAM-5′-TCC TAC CCC AAT TTC CAA TGC-3′, reverse, 5′-TGA ATT GGA TGG TCT TGG TCC T-3′, probe, FAM-5′-CAG ATA AGC TGG AGT CAC AGA AGG AGT GG-3′-TAMRA; MIP2, forward, FAM-5′-TGA CTT CAA GAA CAT CCA GAT CTT-3′, reverse, 5′-CTT GAG AGT GGC TAT GAC TTC TGT CT-3′, probe, FAM-5′-TGA CGC CCC CAG GAC CC CA-3′-TAMRA; β-actin„ forward,FAM-5′-AGA GGG AAA TCG TGC GTG AC-3′, reverse,5′-CAA TAG TGA TGA CCT GGC CGT-3′, probe, FAM-5′-CAC TGC CGC ATC CTC TTC CTC CC-3′-TAMRA. For TSLP and Il-33, TaqMan primer and probe mixtures were purchased from Applied Biosystems (IDs: Mm01157588-m1 and Mm00505403-m1, respectively). Beta-actin was used as endogenous reference gene to normalize sample variation and relative expression was calculated by the change-in-threshold (–ΔΔC_T_) method as described (36). Expression in ears and dLn was determined with a TaqMan real-time PCR assay using a CFX96 PCR system (Bio-Rad). PCR data were reported as the relative increase in mRNA transcripts using the levels of β-actin mRNA for normalization.

### MC quantification

MC numbers in skin were evaluated in anesthetized RMB mice treated with either PBS or DT by placing them on a custom-designed heated platform. A metal ring was clued on the ear and filled with echography gel aquasonic 100 parker allow immersion of a X20/0.95NA dipping objective (Olympus). Imaging was carried out using an upright two-photon microscope (DM6000B, Leica Microsystems) with excitation provided by a Ti-sapphire laser (Coherent) tuned at 900–950 nm. We detected fluorescence with four nondescanned detectors (Leica Microsystems). Typically, we collected images from 10 to 15 *z*-planes, spaced 5 μm apart, every 5 min for up to 90 min. To evaluate degranulated MC sections of ear sections of nongrafted and allograft transplanted tissues were embedded in paraffin and stained with toluidine blue as described (37) and the percentage of degranulated MCs was evaluated.

### General experimental design and statistical analysis

For animal experiments, littermates were used and randomly assigned to PBS or DT treatment groups. All experiments were conducted at least three times as indicated in the figure legends. Statistical analysis was performed using GraphPad software Prism 6.0 and statistical tests are indicated in the figure legends. Data are presented as the means ± SEM, and *P-*values of < 0.05 (1 ${}_{{}^{*}}$, #, §), 0,01 (2 ${}_{{}^{*}}$, #, §), 0,001 (3 ${}_{{}^{*}}$, #, §), 0,0001 (4 ${}_{{}^{*}}$, #, §) were considered significant.

## Results

### Mast cells accelerate graft rejection in the skin

**Figure 1:**
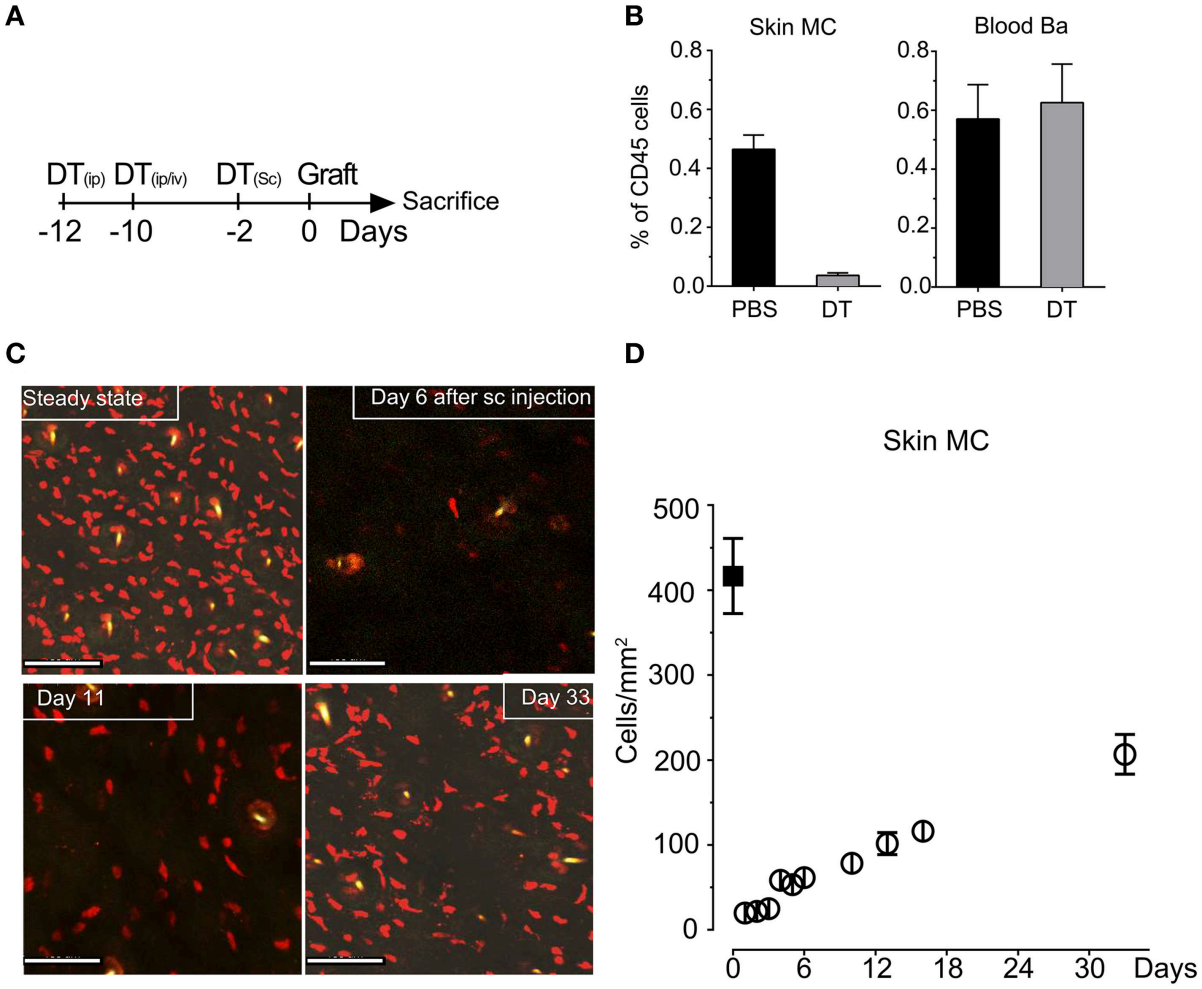
Injection of DT in RMB mice induces selective long-term depletion of MC in the skin of RMB mice. **(A)** Schematic presentation of the protocol of DT injection used throughout the study to allow selective depletion of MC in the ear skin and full replenishment of Ba. **(B)** Analysis of the fraction of skin tdT/c-kit positive MC and blood tdT/CD49b-positive Ba amongst CD45 positive cells by flow cytometry. Bars represent means ± SEM obtained from at least 3 independent experiments (total *n* = 6 to 8 mice/group). **(C)** Long-term depletion of MC in the skin. Left panel shows representative confocal images of tdT positive MC in the skin after PBS or DT treatment at the days indicated. Scale bars: 150 μm. **(D)** Quantitative analysis of skin mast cells in confocal images at the indicated times after depletion (empty circles, black squares represent numbers in untreated RMB mice). Data represent means plus/minus SEM from at least 8 high power fields counted from 3 independent experiments.

To investigate MC function in skin grafting, we took advantage of our RMB mice, which allow diphtheria toxin (DT)-mediated ablation of MCs and basophils as well as their visualization via the bright red td-Tomato (tdT) fluorescent protein (29). We initially verified the efficient depletion of tdT-positive MCs in the skin after systemic and local injections starting 12 days before the intended surgery according to the protocol depicted in Figure 1A to allow basophils replenishment. Analysis of the fraction of tdT/c-kit positive MCs isolated form the ear skin at the day 0 showed a >95% depletion while at this time point tdT/CD49b-positive blood basophils were completely replenished (Figure 1B) as described before (29) generating *de facto* MC-only depleted mice. Follow-up quantification using confocal imaging confirmed that MCs remain substantially depleted up to D11 (<20%); even at D33 less than half of the compartment had been replenished in the skin (Figures 1C,D).

**Figure 2:**
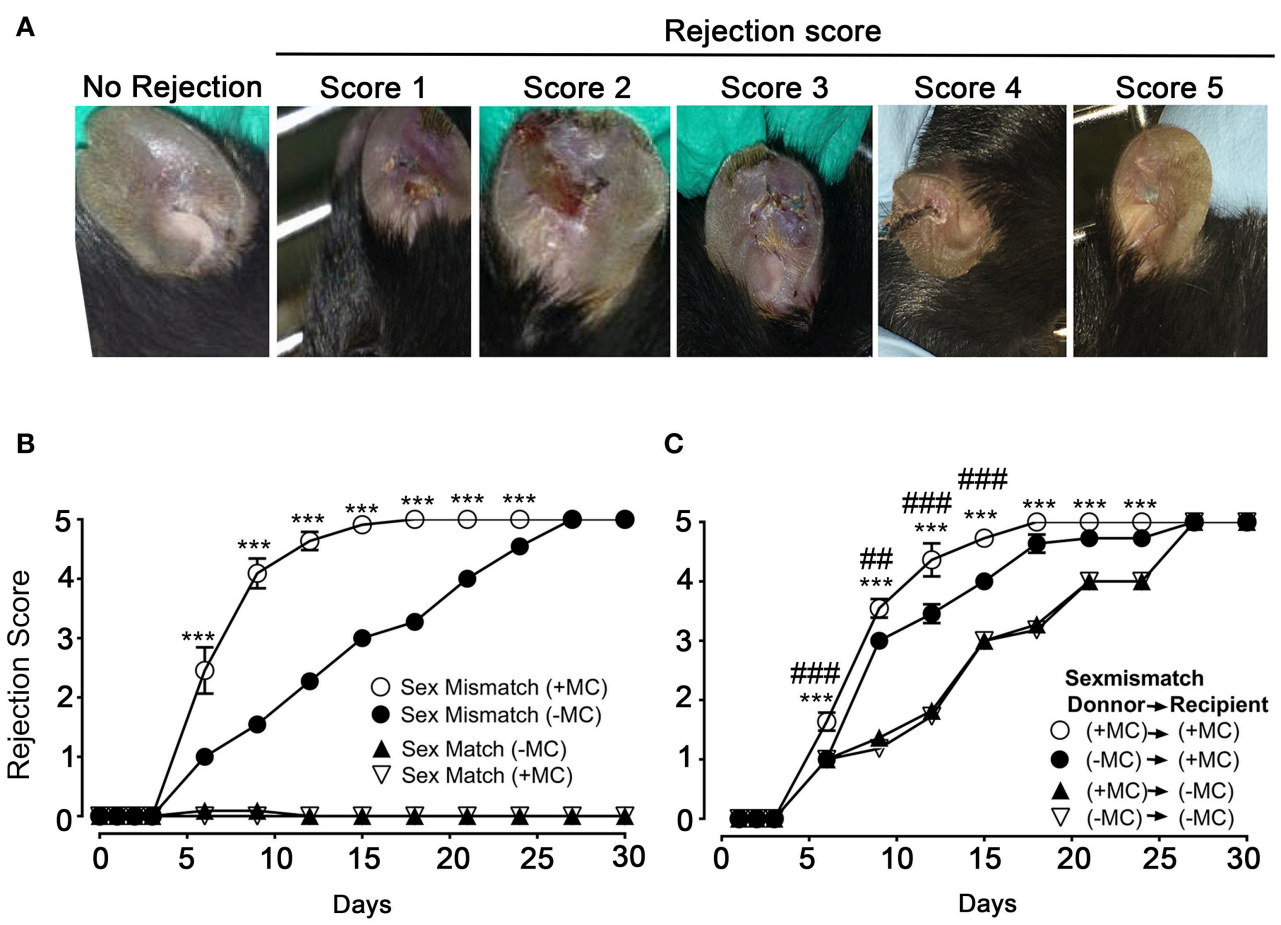
The presence of mast cells accelerates graft rejection in a sex-mismatch MHC ClI-restricted minor transplantation antigen donor ear skin model. **(A)** Representative images of progressive rejection scores as detailed in Materials & Methods and used for evaluation are shown. **(B)** Kinetic analysis of rejection in sex-matched and sex-mismatched animals treated with PBS- (+ MC) or DT (- MC) in both host and donor mice as indicated. **(C)** Kinetic analysis of rejection in sex-mismatched animals treated with PBS- (+ MC) or DT (- MC) in either donor, host or both as indicated. Data in B and C represent means ± SEM of rejection scores from at least 4 independent experiments (total *n* = 8 to 10 mice/condition. *compares PBS-treated vs. DT-treated (MC-deficient) mice in both host and donor, ^#^ compares PBS-treated vs. DT-treated (MC-deficient) mice in donor only. Differences between other groups of mice were not statistically significant. Statistic was analyzed using one- way ANOVA test using the nonparametric Bonneferoni multiple comparisons test (comparing all the groups).

We next undertook skin allograft surgery using the sex-mismatch H-Y minor transplantation antigen donor ear skin model (26, 27). An example of our scoring strategy of graft-survival is shown in Figure 2A. Kinetic experiments in the presence or absence of MCs in both donor and host skins were performed (Figure 2B). While sex mismatched ear skin graft rejection occurred rapidly (median rejection (MR) ~6 days) in the presence of MCs (PBS injected mice), there was a marked delay (MR ~15 days) in the absence of MCs (DT injected mice). No differences were seen in the case of sex matched grafts, which were readily accepted whether MCs were present or not suggesting that MC were not essential for syngeneic graft acceptance (Figure 2B). We also analyzed whether MCs in either host or donor alone could influence the rejection profile. Depletion of MCs in the host alone (male PBS → female DT) did not significantly impact the delay of rejection as compared to the situation where both graft and donor (male DT → female DT) were depleted of MCs indicating a dominant role of the host MCs (Figure 2C). By contrast, only a slight delay in rejection was observed when MCs were present in the host but absent in the donor's skin (male DT → female PBS), as rejection reached its plateau phase at later time points (D18 vs. D21) indicating that graft MCs can also somewhat contribute to the acceleration of rejection (Figure 2C). Taken together these data show that MCs significantly accelerate graft rejection with a major contribution exerted by host MCs in recipient ear and a minor contribution by donor ear skin MCs.

### MCs impact the immune cell infiltrate in the ear and the draining lymph nodes after grafting

**Figure 3:**
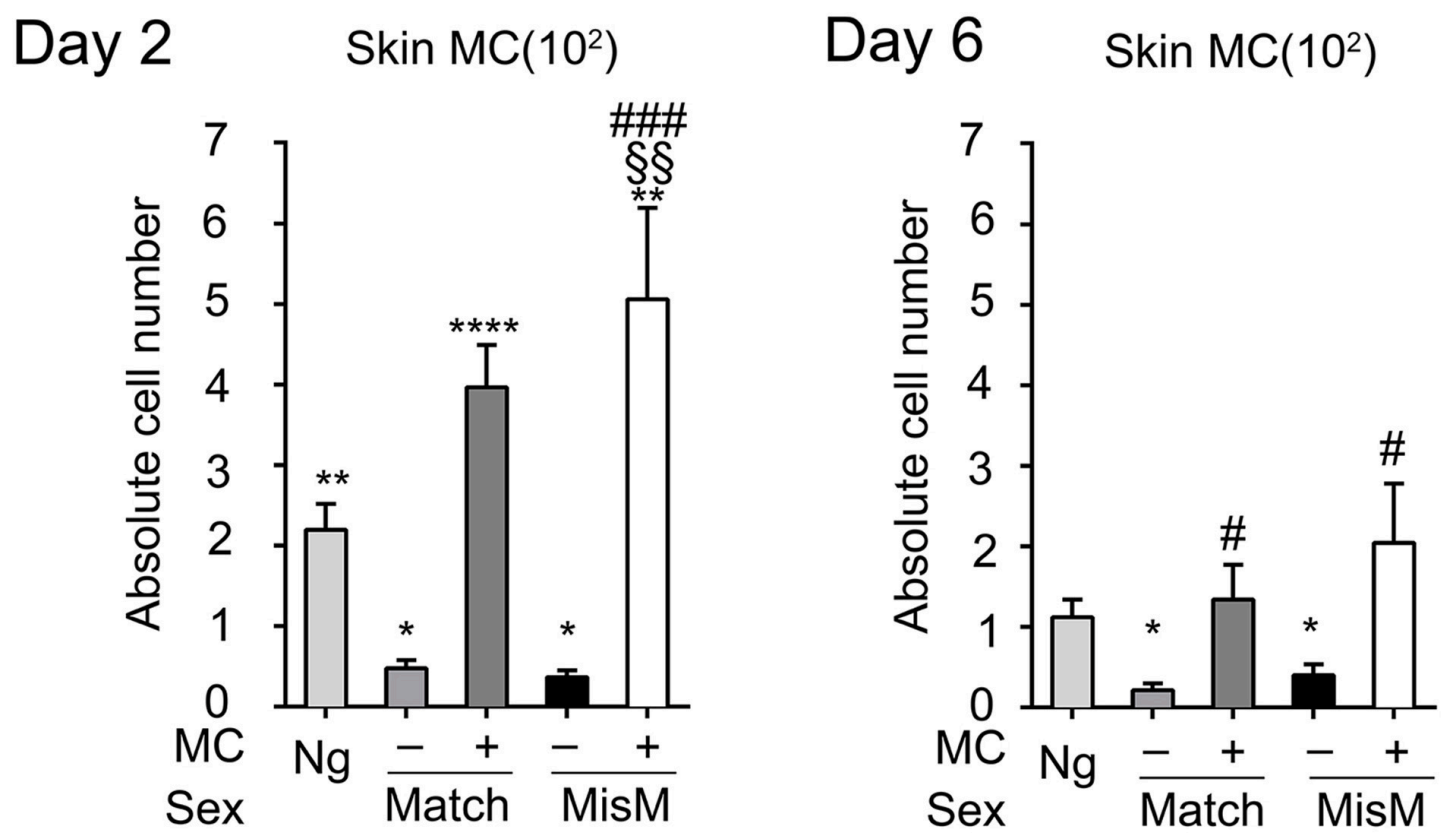
Mast cells transiently increase after grafting under both matched and mismatched conditions. Analysis of skin tdT/c-kit positive MC in nongrafted and grafted tissue as indicated. Numbers are absolute numbers of counted MC per ear pair in the indicated conditions following tissue extraction and flow cytometry analysis as detailed under Materials & Methods. Data represent means ± SEM from at least 4 independent experiments (total *n* = 8 to 10 mice/condition. *compares nongraft vs. graft conditions, ^#^ compares mast cell sufficient PBS (+ MC) vs. MC-deficient DT (-MC) treated mice, ^§^compares matched vs. mismatched conditions using two-way ANOVA test using the nonparametric Bonneferoni multiple comparisons test (comparing all the groups).

As MCs have a major impact in the graft response, we analyzed whether their numbers changed after grafting using flow cytometry analysis following tissue extraction. We chose D2 and 6 after grafting corresponding, respectively to the phase just preceding visible rejection as well as the exponential phase where rejection became maximal (see Figure 2B). Figure 3 shows that MC numbers significantly increased at D2 in both matched and mismatched grafts as compared to non-grafted tissue supporting early MC recruitment and/or proliferation at the inflammatory site. No significant differences were seen between matched and mismatched grafts suggesting that it occurred independent of the status of the graft. At D6 the MC decreased again rejoining MC numbers in steady state tissue both in matched and mismatched grafts. This supports a transient increase of MC infiltration triggered at the initiation of the inflammatory reaction after grafting. As expected, MC numbers were and remained barely detectable in ears from DT-treated mice.

**Figure 4:**
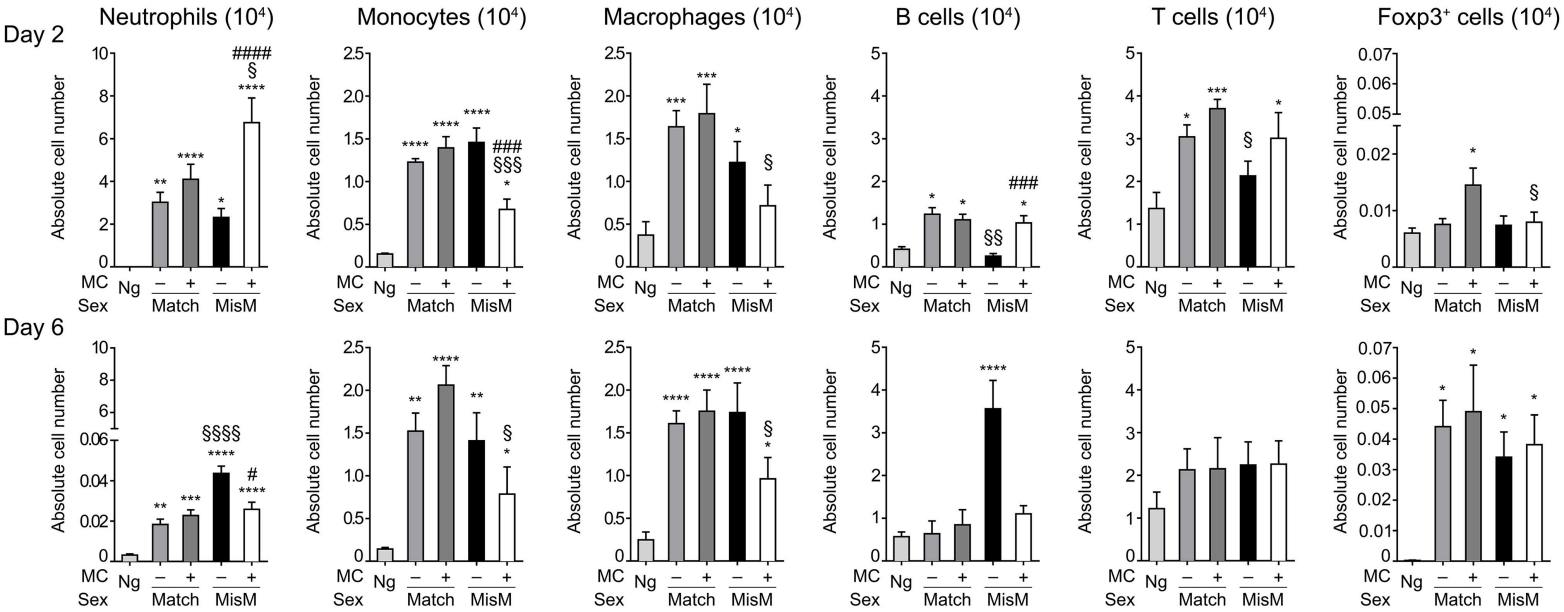
Marked early neutrophil infiltration in ear tissue after grafting is majored under mismatch conditions in the presence of MC. Analysis of neutrophils, monocytes, macrophages, B cells, T cells, and Foxp3-positive regulatory cells in nongrafted and grafted ear tissue at day 2 and 6. Numbers represent absolute numbers of counted cells per ear pair in the indicated conditions following tissue extraction and flow cytometry analysis as detailed under Materials & Methods. Data represent means ± SEM from at least 4 independent experiments (total *n* = 8 to 10 mice /condition. *compares nongraft vs. graft conditions, ^#^ compares PBS- (+ MC) vs. DT (–MC) treated mice, ^§^compares sex-matched vs. sex-mismatched conditions using two-way ANOVA test using the nonparametric Bonneferoni multiple comparisons test (comparing all the groups).

As MCs are known to drive immune cell recruitment during inflammation (25, 38), we analyzed the immune cells in the ear using flow cytometry in MC-sufficient or MC-deficient mice (Figure 4). We focused on neutrophils, monocytes and macrophages for the myeloid lineage as well as B and T cells for the lymphoid lineage. In addition, Foxp3 positive regulatory cells were analyzed using RMB mice crossed with Foxp3-eGFP mice. Most strikingly, while essentially no neutrophils were detected in non-grafted tissue, at D2 we found a marked and significant increase of infiltrated neutrophils both in matched and mismatched grafts (Figure 4, D2 panel neutrophils). The increase in neutrophils was significantly majored in mismatched grafts in the presence of MCs while in matched grafts no differences were seen between MC-sufficient or MC-deficient mice. Previous control experiments conducted in RBM mice had already shown that treatment with DT did not affect neither blood neutrophil numbers at steady state nor their infiltration in an inflammatory model promoting neutrophil infiltration in a manner that was independent of DT treatment (29). Furthermore, the increase in neutrophils was transient as their numbers strongly declined (between 50 and 200 fold) at D6 independent of whether MCs were present or not in matched and mismatched ear grafts (Figure 4, D6 panel neutrophils). However, the ratio of neutrophil infiltration reversed at D6 being now higher under mismatch conditions in the absence of MC. They also were more elevated as compared to match conditions supporting an active role of neutrophils in the inflammatory response in allogenic grafts and an active role of MCs in their recruitment.

We also observed a sizable infiltration (3 to 15-fold) of monocytes and macrophages at D2 both in matched and mismatched grafts as compared to non-grafted tissue (Figure 4, D2 panels monocytes and macrophages). In mismatched grafts, while the MC status did not significantly impact macrophage infiltration, the appearance of monocytes was decreased in the presence of MC in sex-mismatched grafts supporting a protective function. For both monocytes and macrophages the presence of MCs led to a decrease of both populations under mismatch vs. match conditions supporting a possible role in repair functions. This profile remained essentially the same at Day 6 with no major changes in total numbers (Figure 4, D6 panels monocytes and macrophages).

B cell recruitment to local skin grafts increased at D2 except for mismatched conditions in the absence of MC where it was less pronounced (~2-fold; Figure 4, panel B cells). The recruitment was higher in the presence of MC in mismatched grafts while they were similar in the case of matched grafts. At D6 all B cells returned to baseline levels reaching levels of non-grafted tissue except for the mismatched graft where a strong increase in the absence of MC was observed supporting a regulatory role of MCs under these conditions (Figure 4, panel B cells). Concerning T cells, their numbers also increased in the graft as compared to non-grafted tissue at D2 and stabilizing at D6 (Figure 4, panel T cells). Under mismatch conditions in the absence of MCs we observed, however, a slight delay at D2 as compared to matched conditions. The difference to the presence of MCs was, however, not statistically significant (Figure 4, panel T cells). Concerning Foxp3-positive cell recruitment (Figure 4, panel Foxp3) it was minor at D2, and appeared elevated only in the presence of MCs in the case of matched graft, but not mismatched grafts. However, at D6 a large increase in Foxp3-positive infiltration was noted with no significant differences being apparent between the various categories.

**Figure 5:**
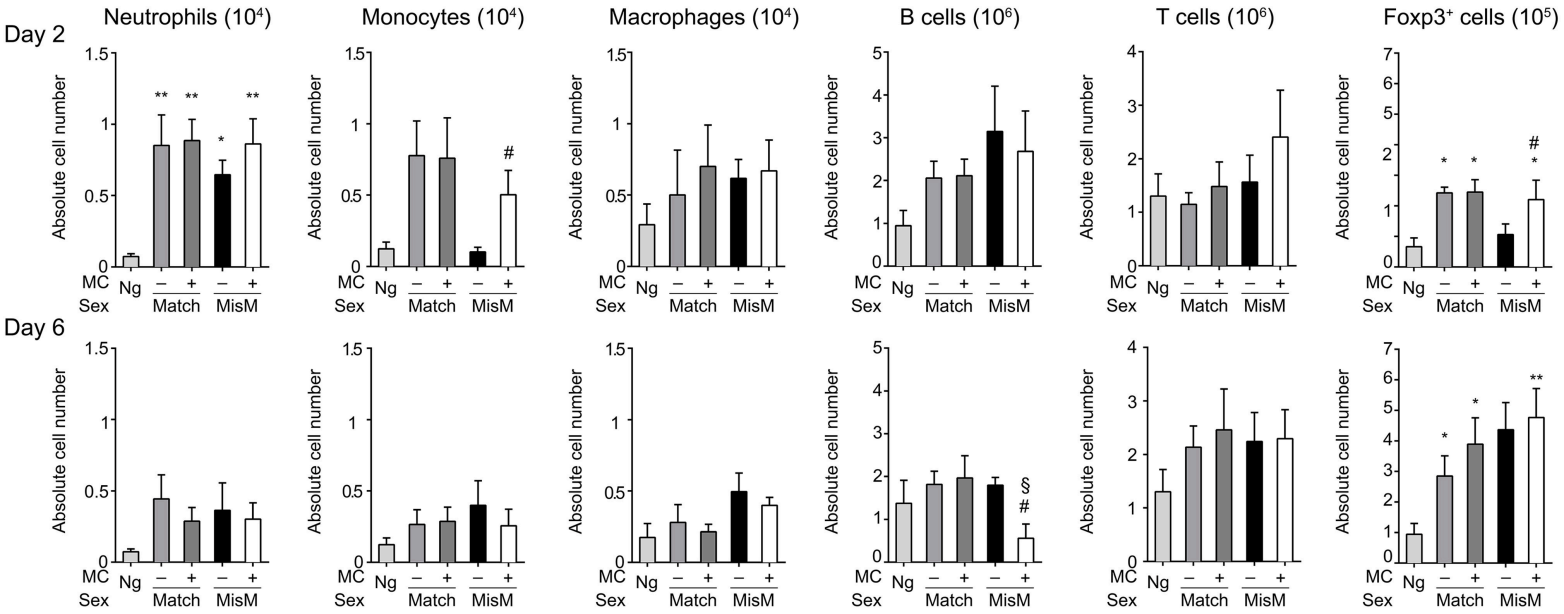
Mast cells induce some early transient changes in monocyte and Foxp3-positive cell infiltration in draining lymph nodes after grafting under mismatch conditions. Analysis of neutrophils, monocytes, macrophages, B cells, T cells, and regulatory T cells in draining lymph nodes of nongrafted and grafted draining lymph nodes (cervical in the neck region) at day 2 and 6. Numbers represent absolute numbers of counted cells in pooled lymph nodes in the indicated conditions following tissue extraction and flow cytometry analysis as detailed under Materials & Methods. Data represent means ± SEM from at least 4 independent experiments (total *n* = 8 to10 mice/ group. *compares nongraft vs. graft conditions, ^#^ compares PBS- (+ MC) vs. DT (–MC) treated mice, ^§^compares sex-matched vs. sex-mismatched conditions using two-way ANOVA using the nonparametric Bonneferoni multiple comparisons test (comparing all the groups).

We also analyzed draining cervical lymph nodes (dLN) after graft transplantation (Figure 5). In the dLN MCs did not influence the immune cell infiltrate under sex-matched conditions as no significant differences between the presence or absence of MCs were observed neither at D2 nor at D6 with a few exceptions. The absence of MCs under mismatch conditions led to a reduced accumulation of monocytes and Foxp3-positive cells (Figure 5, panels, monocytes and Foxp3). These differences were lost at D6. Concerning B cells, while no differences were seen at D2, we found a relative deficiency in the presence of MCs at D6 between matched and mismatched conditions as well as under sex-mismatched conditions when comparing MC-sufficient and MC-deficient mice (Figure 5, panel B cells).These data indicate that the impact of MC on the dLN is relatively minor, albeit some influence on monocyte, Foxp3-positive cell, and B cell populations can be noted.

Taken together, we found that MCs promote a highly prominent local infiltration of neutrophils into both syngenic and allogenic ear skin grafts at D2, which essentially disappeared at D6. In syngenic grafts this infiltrate was independent of the MC status, while in allogenic grafts their infiltration was clearly MC-dependent being two-fold higher than in matched grafts. Some differences were also seen for some other populations, however, albeit their differences in numbers were less when compared to the early neutrophil infiltration.

### MC promote enhanced production of several cytokines and chemokines in the early phase after grafting

**Figure 6:**
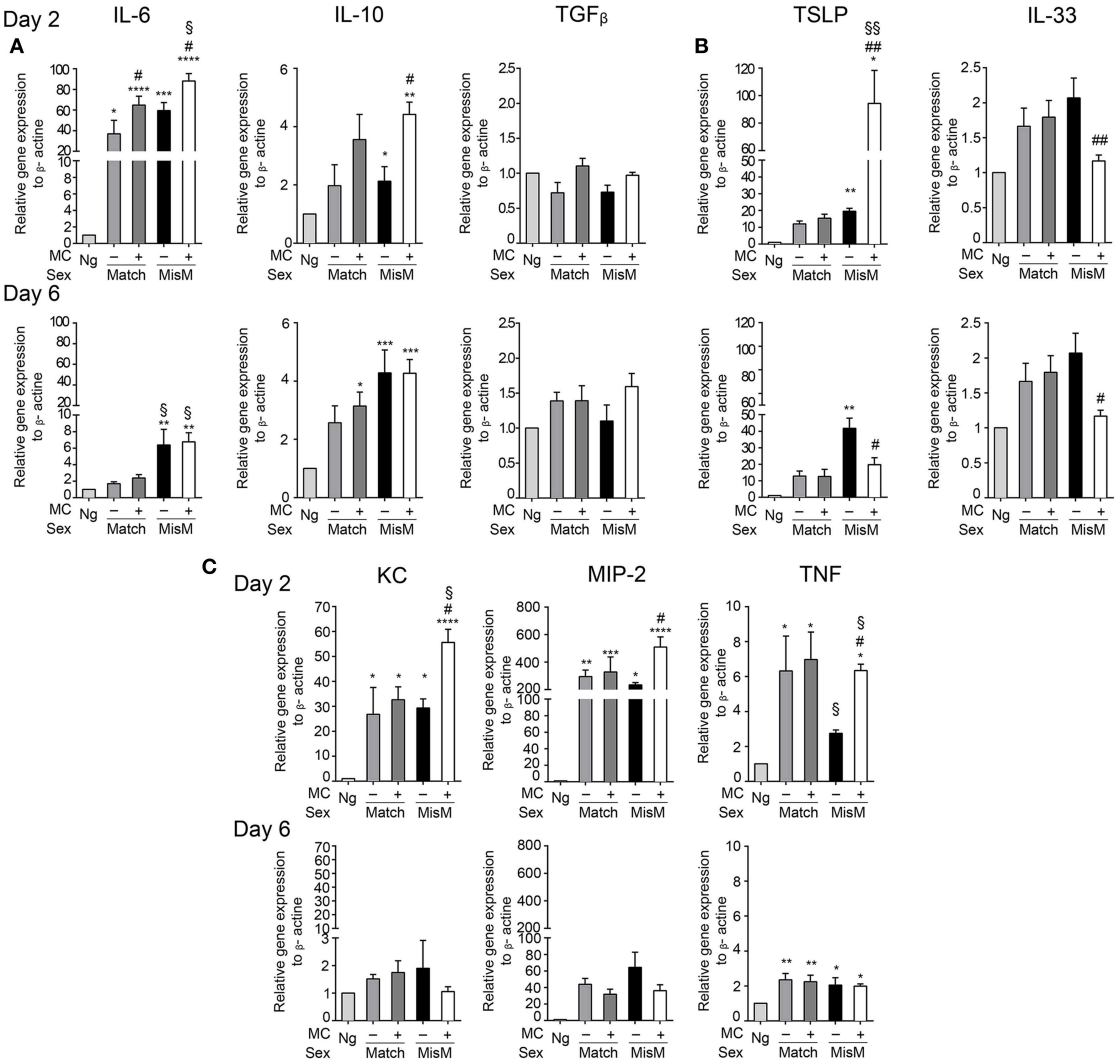
Grafting is associated with significant MC dependent cytokine mRNA levels involved in graft rejection, MC activation and neutrophil chemotaxis. Analysis of indicated cytokines and chemokine mRNA levels in nongrafted and grafted ear tissue at day 2 and 6 using qRT-PCR. mRNA levels of chemokines/cytokines and beta actin, respectively, were determined after extraction from ear pairs and the ratio in nongrafted tissue was arbitrarily set to 1 and compared to the ratios obtained in grafted tissues as indicated. Data in **(A–C)** represent means ± SEM from at least 4 independent experiments (total n = 8 to 10 mice /group. * compares nongraft vs. graft conditions, ^#^ compares PBS- (+ MC) or DT (- MC) treated mice, ^§^ compares sex-matched vs. sex-mismatched conditions using two-way ANOVA using the nonparametric Bonneferoni multiple comparisons test (comparing all the groups).

To assess how MCs could influence the graft-associated inflammatory response, we monitored several pro-and anti-inflammatory chemokines and cytokines focusing on those expressed locally in the graft area using qRT-PCR (Figure 6). They include cytokines described for their role in allograft rejection (IL6, IL10, TGFβ) (39–41), their role in MC activation (IL33, thymic stromal lymphopoetin or TSLP) (32, 42) or neutrophil chemotaxis and activation (MIP2, KC, TNF) (43). Concerning graft-relevant cytokines, at D2 we observed a significant increase in IL6 and IL10 in grafted vs. non-grafted tissues that was slightly favored by MCs in the case of IL6 both under matched and mismatched conditions, while TGFβ mRNA levels were not different (Figure 6A). At D6, IL6 mRNA levels returned to non-grafted baseline levels under matched conditions. Under mismatch conditions, they remain elevated, albeit at lower levels, independent of the presence of MCs. Concerning IL10 and TGFβ, levels were comparable to those at D2.

Concerning the MC-activating cytokines at D2, we found a strong increase in TSLP compared to non-grafted tissue under both match and mismatch conditions (Figure 6B). This increase was further strongly majored in the presence of MCs under mismatch conditions (~80 fold). At D6, levels remained elevated under matched conditions independently of the MC status. Under mismatch conditions TSLP further increased in the absence of MCs, but decreased markedly in their presence with level being significantly lower than in the absence of MCs (Figure 6B). This suggest that TSLP is an important inflammatory cytokine under mismatched conditions that is particularly boosted by MCs during the initial phase of grafting. IL33 showed a tendency to increase after grafting especially at D6, while this was not evident at D2 (Figure 6B). Yet, we saw some differences between the presence or absence of MCs. The latter supported MC-dependent IL33 production in the early phase, while in the later phase at D6 this was reversed.

For the neutrophil attracting chemokines/cytokines (Figure 6C), we observed a strong induction in the early phase for KC, MIP2, and TNF both under matched and mismatched conditions. While no differences were seen between the absence or presence of MCs under matched conditions in this early phase, MCs clearly supported KC, MIP2, and TNF mRNA induction under mismatch conditions, in accordance with the amount of neutrophils recruited locally. At D6, levels declined strongly for KC (reaching values of non-grafted tissue), moderately for MIP 2 and slightly for TNF and the noted MC-dependent differences under mismatch conditions disappeared (Figure 6C).

Together these data indicate that the presence of MC significantly enhances several cytokine and chemokine mRNA levels especially during the early phase of allografting supporting an important contribution to the acute inflammatory response. The most striking differences were observed for the MC activating cytokine TSLP as well as for the neutrophil attracting chemokines/cytokines KC, MIP-2, and TNF under mismatch conditions.

### Neutrophils are important actors in early graft rejection

**Figure 7:**
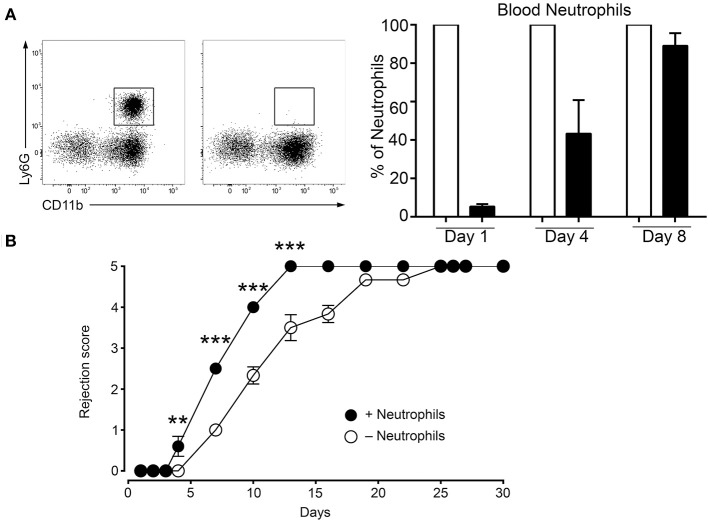
Deletion of neutrophils delays rejection. **(A)** C57BL/6N mice were treated twice at 16 h before grafting and at Day 4 after grafting i.p. with 200 μg of anti-neutrophil (NIMP-R14) or isotype control antibodies. Representative images of the assessment of neutrophil depletion shows dot blots of the neutrophil population in control and anti-neutrophil treated animals at indicated times. **(B)** Kinetic analysis of rejection in sex-mismatched animals treated with control and anti-neutrophil antibody in the host as indicated. Data represent means ± SEM of rejection scores from at least 4 independent experiments (total *n* = 8 to 10 mice/condition) using one-way ANOVA using the nonparametric Bonneferoni multiple comparisons test (comparing all the groups).

The data above show that the MC initiated inflammatory response under mismatch conditions leads to neutrophil infiltration that is largely increased as compared to syngenic grafts (Figure 4, panel neutrophils). Therefore, we sought to determine whether the MC-promoted neutrophil-driven inflammation directly contributes to graft rejection. This was analyzed by undertaking neutrophil depletion experiments. Preliminary experiments established that, as reported before (33), neutrophils were essentially depleted 24 h after an i.p. administration of anti-neutrophil Ab, but reappeared at D4 (Figure 7A). We therefore adopted a protocol requiring a second injection of to maintain depletion. Applying this depletion protocol to a male to female graft our data followed for 9 days show that neutrophil depletion significantly retards rejection, indicating the contribution of neutrophils to acceleration of graft rejection (Figure 7B).

### Mast cell degranulation is a key event for early graft rejection

**Figure 8:**
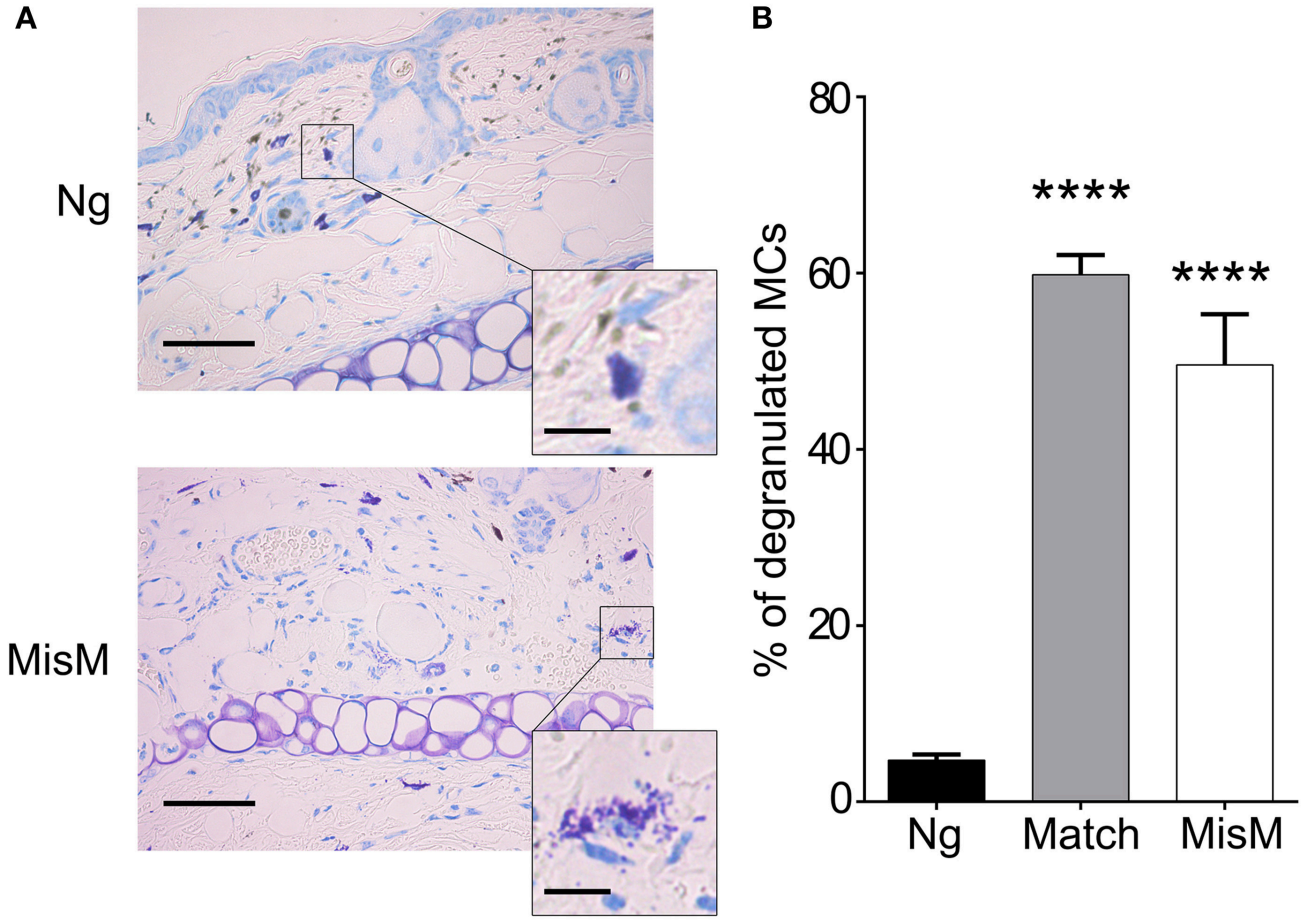
MCs degranulate after grafting both under matched and sex-mismatch conditions **(A)** Representative photomicrographs of ear skin from nongrafted and sex-mismatched grafted tissue stained with toluidine blue. Degranulated MC visible in allogenic grafts are indicated by arrows. The inset show a high magnification picture of a nondegranulated and a degranulated MC **(B)** Quantitative evaluation of degranulated MC in nongrafted as well as matched and sex-mismatched grafted tissues. Scale bar: 100 μm; insert scale bar: 10 μm. Data represent the percentage of degranulated MC (means ± SEM) present 8 high power fields counted from at least three independent experiments using the Student's t test. No significant differences were observed between matched and mismatched conditions.

**Figure 9:**
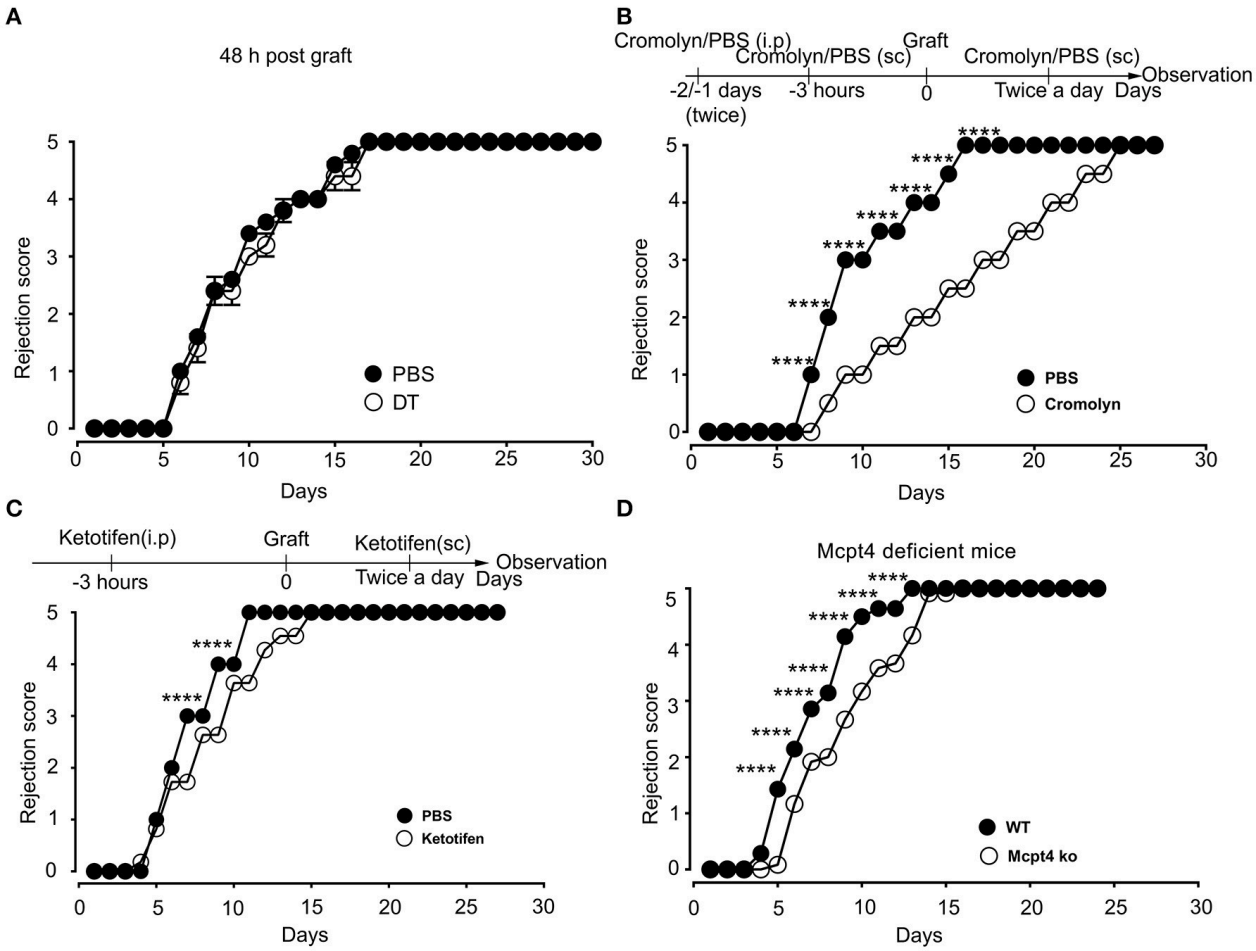
Blockade of mast cell degranulation or certain MC mediators released by degranulation delays rejection. **(A)** Kinetic analysis of rejection in sex-mismatched animals treated with PBS (+ MC) or 1 μg DT (–MC) injected sc into the ear pinnae 2 days after allografting to allow an initial MC-mediated acute inflammatory response. Data represent means ± SEM of rejection scores from 4 to 5 independent experiments (total *n* = 8 to 10 mice/condition. from one-way ANOVA using the nonparametric Bonneferoni multiple comparisons test (comparing all the groups). **(B)** C57BL/6N mice were treated i.p. with 100 mg/kg of Cromolyn sodium or PBS solvent 30 min before male to female grafting as indicated. Treatment was continued every day after grafting until sacrifice and the rejection profile was monitored. Data show the quantitative analysis of rejection in sex-mismatched animals treated with solvent and cromolyn as indicated. Data represent the means ± SEM of rejection scores from 4 to 5 independent experiments (total *n* = 8 to 10 mice/condition. from one-way ANOVA using the nonparametric Bonneferoni multiple comparisons test (comparing all the groups). **(C)** C57BL/6N mice were treated i.p. with SEM from two-way ANOVA test **(B)** C57BL/6N mice were treated i.p. with (32 mg/kg) of the H1R antagonist Ketotifen or solvent DMSO before male to female grafting as indicated. Treatment was continued every day after grafting until sacrifice and the rejection profile was monitored. Data represent the means ± SEM of rejection scores from 4 to 5 independent experiments (total *n* = 8 to 10 mice/condition. from one-way ANOVA using the nonparametric Bonneferoni multiple comparisons test (comparing all the groups). **(D)** Male to female grafting was performed in *MCPT4–/–* or C57BL/6N mice and the rejection profile was monitored. Data represent the means ± SEM of rejection scores from 4 to 5 independent experiments (total *n* = 8 to 10 mice/condition. from one-way ANOVA using the nonparametric Bonneferoni multiple comparisons test (comparing all the groups).

Previously, De Vries et al reported that degranulation by MCs could break an established tolerant state in an experimental model where skin grafts were rendered tolerant albeit the precise mechanism was not investigated (44). Given that the presence of MCs strongly affected the early inflammatory response, we surmised that MCs might exert their pro-inflammatory activity during the early phase of allografting by degranulation. Thus, we examined whether MCs in the grafted tissue exhibited a degranulated phenotype after allografting. As depicted in Figure 8, MCs showed a significantly increased degranulated phenotype both under conditions of match and sex-mismatch compared to non-grafted tissue. As this degranulation was observed within 6 h after allografting, we examined whether it is their early implication in the inflammatory response during grafting, which is responsible for the observed delay in rejection. To test this, we took advantage of RMB mice, which allow depletion of MCs after the acute inflammatory phase (i.e., at D2 after allografting). As shown in Figure 9A, when MCs were depleted locally by a subcutaneous injection into the ear pinnae 2 days after grafting, the delay in rejection in the absence of MCs was abolished. This supports that MCs enhance rejection during the acute inflammatory phase, likely due to their ability to release many vasoactive and proinflammatory compounds by degranulation. To test this directly, we analyzed whether blocking MC degranulation or certain MC degranulation products could impact the rejection profile. As shown in Figure 9B treatment with cromolyn, a known MC degranulation blocker (35), before and after grafting led to a marked delay in graft rejection. At the same time neutrophil recruitment was reduced by about 5.5-fold (1.9 ± 0.41 × 10^4^ vs. 10.5 ± 1.48 × 10^4^ neutrophils). This further supports that MC preformed mediator release (i.e., degranulation) contributes to graft rejection through their ability to recruit neutrophils. Treatment of mice with Ketotifen, an antagonist of the histamine type 1 receptor, known to be expressed on the vasculature (34), also induced a slight delay, albeit the effect was less pronounced than with cromolyn (Figure 9C). MCPT4, a MC-released chymase expressed in skin MCs, is a known chemoattractant for neutrophils (45, 46). We therefore performed allografting in MCPT4-deficient mice. Here also, rejection was slightly but significantly delayed in the absence of MCPT4 when compared to wild-type mice (Figure 9D). Again, the delay was less pronounced than after cromolyn treatment (Figure 9B).

Together, these data support that MCs accelerate rejection of allografts by driving an acute inflammatory response after degranulation. The fact that testing of individual granule-contained mediators had a partial effect when compared to the degranulation blocker cromolyn supports that it is likely the combined action of several MC degranulation products, which is necessary to more profoundly delay the graft rejection.

## Discussion

Although adaptive immunity has been recognized and studied for decades for its role in graft rejection, recent studies support also a role of innate immunity (4). Besides orchestrating the adaptive immune response, it can promote a graft inflammatory response that may itself contribute to the initiation of rejection events leading to the destruction of grafted tissue (47). In some occasions it was also reported that innate effectors such as monocytes may be directly involved in the recognition of alloantigen (48). In this study, we have set up a skin allograft model to investigate the functional contributions of MCs to rejection. Skin allografts are at the lower end of graftable tissue even under conditions of immunosuppression due to the pronounced graft-associated inflammatory response and to the low numbers of effector T cells required for rejection (8, 9, 49). To minimize the contribution from other immune effector cells such as NK and B cells we choose a minor transplantation antigen and T cell dependent sex-mismatched allograft protocol (28). The skin was chosen as a graftable tissue since mast cells are highly prominent in skin already at steady state, thus representing a relevant tissue for examining their function [(50), see also Figure 1). Furthermore, previous analysis in this tissue had already shown that mast cell degranulation may impact their capacity to induce a tolerant state (44) supporting that the induced inflammatory response could also impact rejection in the context of primary graft rejection without preconditioning for tolerance. Using this model our findings support a crucial role of MCs in the acceleration of rejection by promoting an early inflammatory response through their degranulation. This led to an important unbalanced neutrophil infiltration known to participate in the initiation of tissue destruction in allografts (5). Therapeutic strategies aimed at the stabilization of MCs using cromolyn that blocks degranulation (51) decreased the rejection rate. This effect was partial when individual MC mediators such as histamine or mast cell chymase MCPT4 were blocked supporting that MCs enhanced rejection through the release of a diverse set of mediators released through degranulation.

MCs are highly prominent inflammatory effectors in the skin accounting for >400 cells /mm^2^ (see Figure 1). Moreover, independent data indicated that MCs could represent central effectors in the graft associated inflammatory response and hence contribute to graft rejection. Indeed, it was reported that MCs readily increase both in acute and chronic grafts in different tissues (15, 52–55). This is also confirmed by our data that show an enhanced presence of MCs in the grafted ears at D2. This increase in MC numbers was transient since at D6 numbers reached again baseline levels. Analysis of their degranulation status clearly showed that rapidly after allografting, a sizable fraction of MCs become activated as revealed by their degranulated phenotype. This was independent of whether the graft was syngenic or allogenic. Presently, the mechanism of activation remains unknown. However, given their large panoply of expressed cell surface receptors and the strong inflammatory response associated with grafting it is likely that multiple activation pathways may be implicated (13). In agreement, we measured a strong increase of TSLP (> 80-fold) and to a minor extend (>2-fold) of IL33 in the case of mismatched grafts. Although, this was MC-dependent these cytokines are likely released by keratinocytes and epithelial cells known to be strong producers of these cytokines in skin-associated inflammatory responses (42). Both cytokines are known to represent important MC activators supporting an amplification loop between tissue-associated inflammatory events and MCs (42). Interestingly the increase in TSLP was particularly prominent under allograft conditions in the presence of MCs supporting that they may be able to distinguish signals after allografting as previously reported for monocytes (48). However, like for the latter the mechanism remains unknown.

One of the important consequences of grafting was the rapid and massive infiltration of neutrophils at D2. Several previous studies showed that neutrophils directly contribute to graft rejection via an innate immune response through their capacity to produce large amounts of oxidative species, myeloperoxidase as well as proteases with cytotoxic effects (5). The influx in neutrophils was highly prominent during the early phase after grafting but transient as their numbers largely dropped by 50 to 200-fold at D6 in transplanted ears. Interestingly, a strong and transient neutrophil infiltration was also observed in matched grafts in a MC-independent manner. Yet, under mismatch conditions their numbers reached significantly higher levels in the presence of MCs again supporting that they may be able to distinguish signals after allografting. The significant recruitment of neutrophils under matched conditions suggests that they may also contribute to graft repair mechanisms by a MC-independent mechanism. Indeed, recent data provided evidence for a contribution of neutrophils to repair in sterile inflammation performing critical tasks such as dismantling injured vessels and creating channels for new vascular regrowth (56). Yet, we also confirmed the role of neutrophils in the acceleration of mismatched graft rejection as their selective Ab-mediated depletion led to a significant delay in this process. Therefore, neutrophil infiltration needs to be tightly controlled to favor repair and limit pro-inflammatory pathologic consequences. In agreement, our data show that in the case of mismatched grafts, neutrophil infiltration further increases as compared to matched grafts by a MC-dependent mechanism. In addition, in mismatched grafts an about five-fold increase in neutrophil infiltration was noticed in the presence of MCs as compared to their absence supporting that it is the unbalanced neutrophil influx, which contributes to graft rejection. The involvement of neutrophils in the rapid rejection supports that this process can at least partially be mediated directly by innate immune cells (MCs and neutrophils) controlling the extent of the inflammatory response. This notion is also supported by our data, which did not reveal significant differences in T cells between the various groups (matched vs. mismatch; absence vs. presence of MCs) at the early time points of rejection up to D6. Likewise, we did not observe major differences in Foxp3-positive cell infiltration except for the early stage at Day 2 where more cells were seen in the presence of MCs. However, it is unlikely that at this stage their presence is due to an adaptive response but seems rather triggered by MC released factors, possibly IL-2 (57). Concerning B cells we rather saw a decrease in the presence of MCs at the later stages (D6) supporting that MCs do not influence the B cell effector response under these conditions. Some differences were also seen for monocytes and macrophages either in the graft or in draining lymph nodes again supporting possible innate functions. However, presently we did not investigate the functional significance of these differences.

Concerning the possible mechanisms by which MCs contribute to the accelerated graft rejection and to the increase in neutrophil recruitment, we analyzed proinflammatory cytokines and chemokines. Our data show that MCs had only a minor effect on graft-associated cytokines at D2 slightly enhancing mRNA levels of graft-associated pro-inflammatory IL6 as well as anti-inflammatory IL10, while no effect was seen for TGFβ. No differential effects were present at the later stages (D6). The fact that the elevation occurs both under matched and mismatched conditions supports that these cytokines do not account for the MC influence on the kinetics of graft rejection. By contrast, when mRNA levels of neutrophil attracting chemokines such as KC (CXCL1), MIP-2 (CXCL2), and TNF were examined we found a strong induction both in matched and mismatched grafts at D2 in agreement with the marked neutrophil infiltration observed at this early time point. Whereas, no differences were seen between matched conditions, we noted a significant MC dependency on the elevation of mRNA levels for all three chemokines/cytokines under mismatch conditions in the early phase again supporting differential effects after allografting. It is possible that the combined action of these three neutrophil attracting cytokines/chemokines contributes to the stronger increase in neutrophil infiltration observed in the presence of MCs in allografts (Figure 4). In addition to chemokines/cytokines induction the ability of MCs to release numerous mediators by degranulation may also contribute to the influx of neutrophils. Clearly, we counted more degranulated MCs after grafting and granule-stored mediators are known to target neutrophils (58). Thus histamine has been known for long as a potent vasodilator enhancing the blood flow within tissues (59). It also has potent vasopermeability activity opening blood vessel as an essential mechanisms necessary for extravasation (60). Furthermore, certain MC specific proteases such as chymase and its murine functional counterpart MCPT4 have been shown to have a direct neutrophil chemoattractant activity both *in vitro* and *in vivo* (45, 46). Likewise, tryptase another abundant protease released by degranulation has also shown effects on vascular permeability and neutrophil chemoattraction (61). However, this effect seems to be indirect and involve the capacity of tryptase to induce IL8 production through the activation of endothelial cells. In agreement with an important role of MC degranulation in the acceleration of rejection our data show that pharmacological blockade of degranulation with cromolyn had a major effect on the kinetics of graft rejection approaching the half-lives achieved with the absence of MCs alone. Although the action of cromolyn on MC degranulation in mice was challenged (62) many other studies reported significant effects with this compound (63–65). It is possible that the time of administration to engender active metabolites may play an important role in different species. Hence degranulation besides fostering repair may also play an important role in rejection. While cromolyn had an important effect, administration of an H1 receptor blocker or experiments conducted in MCPT4-deficient mice showed only a partial effect. This supports that probably several products contained in granules may synergistically act together to produce the biologic effect of MCs in grafting. Some of these effects may be indirect as for example the aforementioned release of MC stimulating cytokines and neutrophil attracting chemokines can also be released by other cells during an inflammatory response.

In conclusion our data show that MCs are important actors in the innate immune response associated with skin allografting. They can accelerate the rejection kinetics, which involves their capacity to degranulate releasing mediators such as histamine and chymase driving an inflammatory response. This inflammatory response leads to an excessive neutrophil influx that contributes to tissue damage and facilitates graft rejection. Hence targeting MC degranulation may not only present a means to reduce ischemia reperfusion injury associated with transplantation (66) but may represent a means to directly decrease allograft rejection.

## Ethics statement

This study was carried out in accordance with the recommendations of #9628-2017040615388469 v5 Ministère de L'enseignement Supérieur, de la Recherche et de L'innovation et Comité D'éthique en Experimentation Animal N°121. The protocol was approved by the ethic committee Paris Nord N°121.

## Author contributions

FNN, EP, SB, RM, and GG performed experiments. FNN, JD, MB, PL, NC, UB, RM, and GG analyzed data, MÅ and GP provided mouse strain and advise. FNN, GG, and UB wrote the manuscript. UB and GG conceived the project.

### Conflict of interest statement

The authors declare that the research was conducted in the absence of any commercial or financial relationships that could be construed as a potential conflict of interest.
